# Effect of hybridized local and charge transfer molecules rotation in excited state on exciton utilization

**DOI:** 10.1038/s41598-021-97229-z

**Published:** 2021-09-03

**Authors:** Gang Sun, Xin-Hui Wang, Jing Li, Bo-Ting Yang, Ying Gao, Yun Geng

**Affiliations:** 1 College of Science, Beihua University, Jilin, 132013 People’s Republic of China; 2 Jilin Provincial Key Laboratory of Straw-Based Functional Materials, Institute for Interdisciplinary Biomass Functional Materials Studies, Jilin Engineering Normal University, Changchun, 130052 People’s Republic of China; 3 Faculty of Chemistry, Northeast Normal University, Changchun, 130024 People’s Republic of China

**Keywords:** Chemistry, Physics

## Abstract

The fluorescent molecules utilizing hybridized local and charge-transfer (HLCT) state as potential organic light-emitting diodes materials attract extensive attention due to their high exciton utilization. In this work, we have performed the density functional theory method on three HLCT-state molecules to investigate their excited-state potential energy surface (PES). The calculated results indicate the T_1_ and T_2_ energy gap is quite large, and the T_2_ is very close to S_1_ in the energy level. The large gap is beneficial for inhibiting the internal conversion between T_1_ and T_2_, and quite closed S_1_ and T_2_ energies are favor for activating the T_2_ → S_1_ reverse intersystem crossing path. However, considering the singlet excited-state PES by twisting the triphenylamine (TPA) or diphenylamine (PA) group, it can be found that the TPA or PA group almost has no influence on T_1_ and T_2_ energy levels. However, the plots of S_1_ PES display two kinds of results that the S_1_ emissive state is dominated by charge-transfer (CT) or HLCT state. The CT emission state formation would decrease the S_1_ energy level, enlarge the S_1_ and T_2_ gap, and impair the triplet exciton utilization. Therefore, understanding the relationship between the S_1_ PES and molecular structures is important for designing high-performance luminescent materials utilizing HLCT state.

## Introduction

Organic light-emitting diode (OLED) was widely studied since the first small molecules device was reported by Tang et al.^1^. The luminescent materials, as the most important component of OLED, were always the focused study by materials scientists^2–6^. One most important requirement of the luminescent materials was a high photoluminescence quantum yield (PLQY). The pure organic materials utilizing hybridized local and charge-transfer (HLCT) state^7–9^ become potential high-efficiency OLED materials considering the low cost, less toxicity, and high exciton utilization.

**Figure 1:**
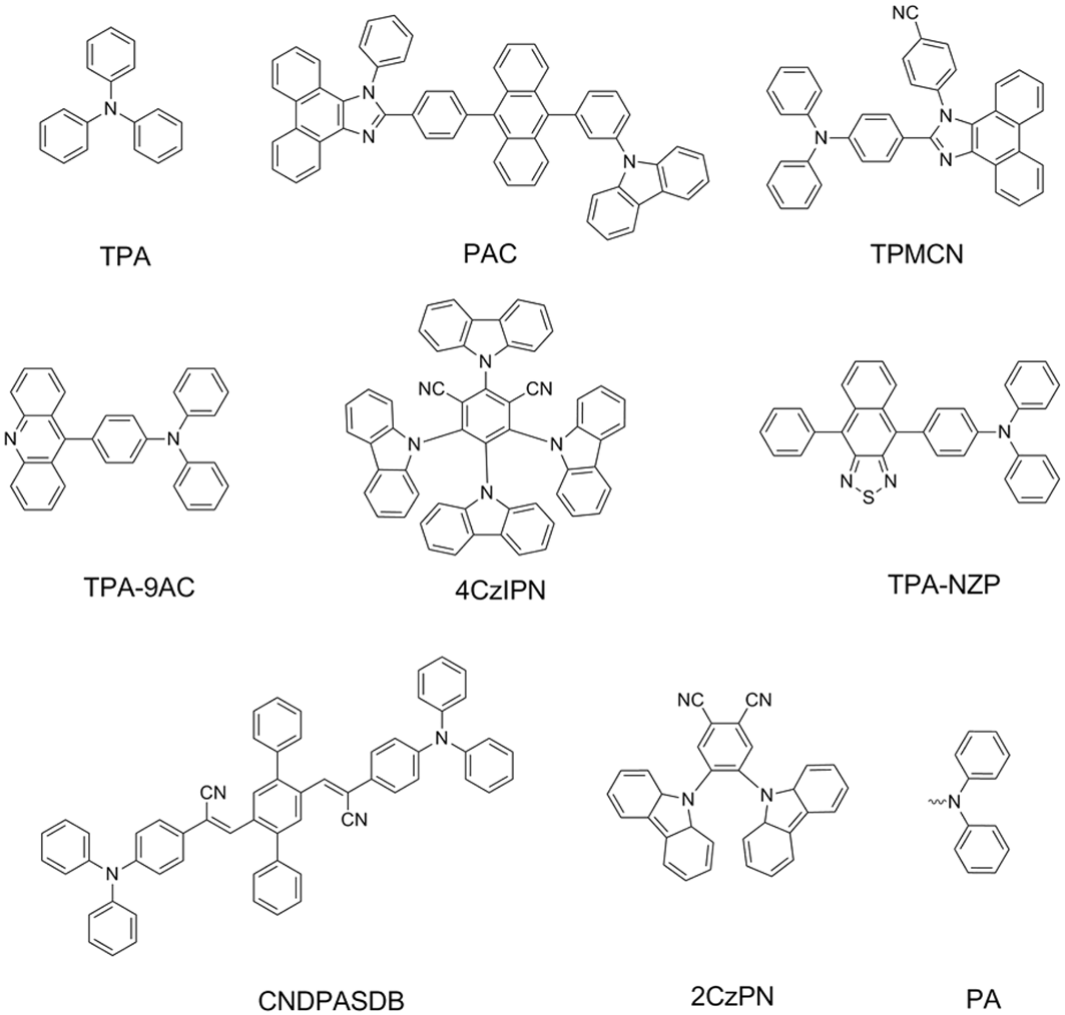
Molecular structures extracted from references.

Numerous highly luminous HLCT-state compounds containing triphenylamine (TPA, Fig. 1) group have been reported^10–15^. For example, in 2014, a series of twisting donor–acceptor (D–A) molecules with high exciton utilization efficiencies and full-color-range emissions were reported^16^. To enhance the proportion of radiative exciton, methods such as constructing orthogonal cyano substituted D–A structure imidazole derivative^17^, designing butterfly-shaped D-A chromophore^18^, modulating a fine proportion of charge-transfer (CT) state and locally excited (LE) state^19^ and choosing an appropriate substituted position, were suggested^20^. Especially, a pure-blue emitting molecule consisting of phenanthroimidazole and phenylcarbazole-substituted anthracene (PAC, Fig. 1) was reported, and the external quantum efficiency (EQE) exceeds 10%^21^. Besides being the emitting materials, the 4-(2-(4-(diphenylamino)phenyl)-1H-phenanthro[9,10-d]imidazol-1-yl)benzonitrile (TPMCN, Fig. 1) was also used as host material with EQE up to 6.3% and exciton utilizing efficiency of 64%^22^. More recently, triphenylamine-acridine (TPA-9AC, Fig. 1) was reported to be a visualization and ultrasensitive fluorescence sensor for diethyl chlorophosphate vapor fast and accurate detection^23^.

Taking the carbazole-dicyanobenzene molecule 4CzIPN (Fig. 1) and triphenylamine-thiadiazole molecule TPA-NZP (Fig. 1) as theoretical models, Pan et al. pointed out different reverse intersystem crossing (RISC) paths (T_1_ → S_1_ for 4CzIPN and T_2_ → S_2_ for TPA-NZP) by comparing the singlet exciton utilization formats using density functional theory calculation^24^. The systematic calculations on accurate description of HLCT state also demonstrate that the ωB97XD or optimally tuned range-separated functional can provide a better description of HLCT state^25,26^.

It is observed that molecules containing TPA group show a sharp emission wavelength dependence on solvent polarity^27–30^. For example, the work done by Xia et al. proposed the explanation that the emissions of cyano-substituted oligo α-phenylenevinylene-1,4-bis(*R*-cyano-4-diphenylaminostyryl)-2,5-diphenylbenzene (CNDPASDB, Fig. 1) were from the LE state in a low-polarity solvent but were from the CT state in the high-polarity solvent^31^. An overall theoretical investigation of TPA-NZP also concluded the excited state changed from HLCT character to complete intramolecular CT with increasing solvent polarities, followed by increased non-radiative decay rate^32^.

Huang et al. also suggested an aggregation-induced emission mechanism that a conversion is from dark twisted intramolecular charge transfer (TICT) excited state to emissive quasi-TICT state because that the restriction of molecular rotation was lower in the amorphous film than in power and crystalline^33^. The fluorescent molecules atomistic molecular dynamics simulation of deposition process showed that the torsion angles between donor and acceptor groups have a broadened distribution around 90 degrees due to the thermal fluctuation and intermolecular interaction^34^. Based on reported compounds (Fig. 1), namely 1,2-bis(carbazol-9-yl)-4,5-dicyanobenzene (2CzPN) and (4CzIPN), the theoretical simulation of bulk amorphous phase also indicated that the torsion angles between donor and acceptor are in a broad distribution, and the RISC is a dynamical process with the varying molecular structures^35,36^.

**Figure 2:**
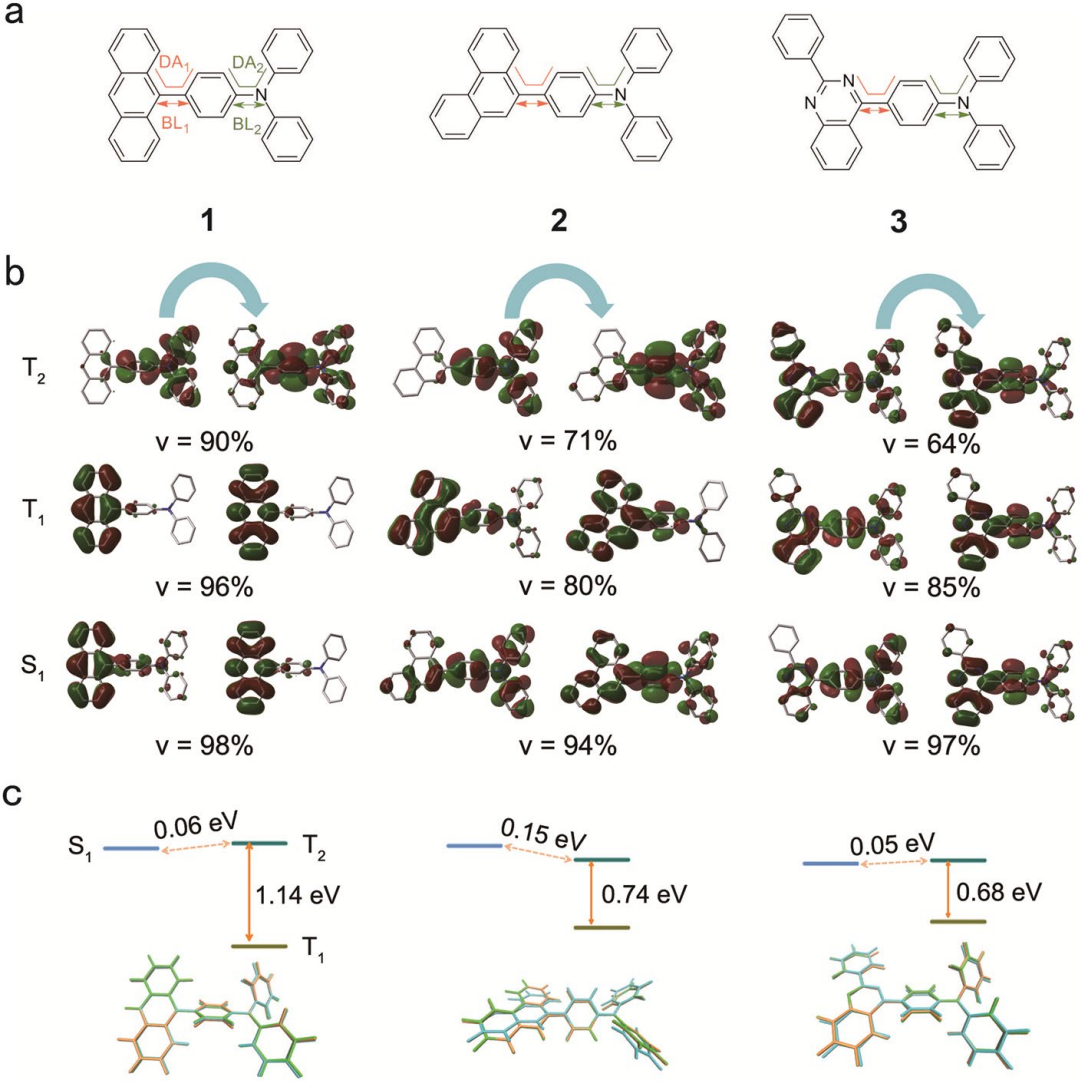
**(a)** Molecular structures of **1**, **2**, and **3**. **(b)** Natural transition orbitals of S_1_, T_1_, and T_2_ excited-state with M06-2X/Def2SVP based on ground-state structures. **(c)** Relative S_1_, T_1_, and T_2_ energy levels with M06-2X/Def2SVP and a structural comparison among S_1_, T_1_, and T_2_ structures. S_1_ (orange); T_1_ (green); T_2_ (cyan). **(b)** Drawn by GaussView 6.0 and Multiwfn programs; **(c)** is drawn by VMD 1.9.1. program.

Ma et al. have proposed the hot-exciton RISC path of the higher triplet state to the single state (T_2_ → S_1_/S_2_) to explain the large proportion of radiative singlet exciton for HLCT-state molecules. However, we wonder whether the excited-state (S_1_, T_1_, and T_2_) energy levels would lead to the variations between S_1_ and T_2_ gaps and affect the RISC process with molecular rotation. Thus, in this work, taking reported HLCT-state molecules (**1**, **2**, and **3**) as examples (Fig. 2a), we have calculated the excited-state potential energy surface (PES) by twisting TPA (Fig. 1) or diphenylamine (PA, Fig. 1) using the time-dependent density functional theory (TD-DFT) method. The calculated results indicate that twisting TPA or PA almost has no influence on T_1_ and T_2_ energy levels, but would affect the S_1_ energy level and the T_2_ → S_1_ RISC process. Therefore, understanding the relationship between the S_1_ PES and molecular structures is important for designing high-performance luminescent materials utilizing HLCT state.

## Computational details

In this work, the ground-state (S_0_) geometries of **1**, **2**, and **3** (Fig. 2a) were optimized at density functional theory (DFT) calculations M06-2X/Def2SVP level. The lowest singlet excited state (S_1_) was obtained with time-dependent density functional theory (TD-DFT) M06-2X/Def2SVP level. Two low-lying triplet excited-state geometries (T_1_ and T_2_) were optimized using the same M06-2X/Def2SVP considering the Tamm-Dancoff approximation (TDA)^37,38^ for avoiding the issue of triplet instability. The corrected linear response (cLR) approach^39^ was employed to obtain the total energies of S_1_, T_1_, and T_2_ excited-state structures. In all calculations, the dimethylsulfoxide (DMSO) solvent under the SMD model^40^ was used to consider the solvent effect because that the high polar DMSO was prone to stabilize the charge-transfer state. To analyze the excited-state (S_1_, T_1_, and T_2_) properties, the vertical singlet and triplet excited states with M06-2X/Def2SVP were calculated based on their S_0_ structures. Besides, the S_1_, T_1_ and T_2_ excited-state potential energy surface (PES) was performed by twisting TPA or PA group with a scan step of 10 degrees. All calculations were accomplished by Gaussian 16 program^41^. The natural transition orbitals, electron and hole analysis and structural comparison among S_1_, T_1_, and T_2_ excited states were obtained by Multiwfn program^42^.

### Consent to participate

All authors agree to participate.

### Consent for publication

All authors agree for publication.

## Result and discussion

### Excited-state properties of 1, 2 and 3

Most reported works explain the high exciton utilization of HLCT-state molecules by analyzing the vertical singlet and triplet excited-state energy levels. In this work, we have optimized the ground-state, singlet, and triplet excited-state structures (S_0_, S_1_, T_1_, and T_2_) and calculated their relatively adiabatic energies. For better describing the excited-state properties, we also illustrate the natural transition orbitals (NTOs) and the associated weights (v) for the singlet (S_1_) and triplet (T_1_ and T_2_) states. However, the v values for some excited states are far away from 100% (in Fig. 2b), and the NOTs are not enough to describe the excited-state properties. Therefore, we further to depict the hole and electron distribution to supplement the NTOs. To analyze the excited states (S_1_, T_1_, and T_2_) properties, the hole and electron distribution were calculated with three methods (CAM-B3LYP, M06-2X, and ωB97XD) based on their S_0_ structures.

The overlap of hole and electron (Sr), separation degrees of hole and electron (t), distance between centroid of hole and electron (D), and overall mean span of hole and electron (H) (in Table S1-S3) were used to describe the quantitative representation of hole and electron distribution, and there are tiny differences in these parameters. However, three methods give similar excited-state characteristics for S_1_, T_1_, and T_2_ states. Figure S1 depicts the electron and hole distribution obtained with CAM-B3LYP, ωB97XD and M06-2X methods. According to the hole and electron distribution, the S_1_, T_1_ and T_2_ states of **1**, **2**, and **3** display main locally excited (LE) and very slight charge-transfer (CT) natures.

We take the plot (Fig. 2b) with M06-2X as an example to discuss the NTOs. As seen in Fig. 2b, the S_1_ states of **1**, **2**, and **3** display main LE and very slight CT natures. For example, the NTOs of **1** mainly locate on the anthracene group and the middle phenyl ring with little CT from the TPA to anthracene group. Both NOTs of **2** and **3** are delocalized over the whole molecules. Such high LE percentages in S_1_ states were beneficial for high fluorescence radiative rate. Similarly, the T_1_ states of **1**, **2**, and **3** also possess main LE and little CT natures. For example, the NTOs of **1** are mainly distributed on the anthracene group, and both **2** and **3** display the NTOs over 2-phenylquinazoline group and middle phenyl ring. As for T_2_, **1**, **2**, and **3** have dominant LE characteristics. **1** has NTOs over the TPA group, and the NTOs of **2** and **3** locate over the whole molecules.

Taking S_0_ as a reference, the relative S_1_, T_1_, and T_2_ energy levels are depicted in Fig. 2c. For all molecules (**1**, **2**, and **3**), the T_2_ is quite close to S_1_ in energy levels, and such a small T_2_ and S_1_ gaps are apt to realize T_2_ → S_1_ reverse intersystem crossing (RISC) path. Besides, compared to the S_1_ and T_2_ gaps, the T_2_ and T_1_ gaps of all molecules (**1**, **2**, and **3**) are relatively large and beneficial to suppress the T_1_ and T_2_ internal conversion, leading to more T_2_ population. The combination of more T_2_ population and negligible T_2_ and S_1_ gaps is advantage for T_2_ → S_1_ RISC, which is consistent with the explanation of the large proportion of radiation singlet exciton for HLCT-state molecules. The **1** and **2** have similar structures, but the T_1_ and T_2_ gap of **1** and **2** are 1.14 eV and 0.75 eV, indicating a slight geometric variation has a huge influence on T_1_ and T_2_ energy gap. Consequently, a careful group combination for designing novel luminescent materials utilizing HLCT state is important.

To quantify the geometric change, we also summarize the typical bond lengths (BL1 and BL2) and dihedral angles (DA1 and DA2) (shown in Fig. 2a and Table S4) in S_0_, S_1_, T_1_, and T_2_ structures. Upon electron excitation from S_0_ to S_1_, T_1_ and T_2_, the DA1 have more noticeable variation (− 45.9 to − 69.0° for **1**) than DA2 (28.8°–38.9° for **1**). Likewise, the BL2 variation between S_0_ and excited state (S_1_, T_1_, and T_2_) is smaller than BL1, and the differences are below 0.03 Å. The BL1 and BL2 variations show the opposite variations to DA1 and DA2. The larger rotations lead to shorter bond lengths between TPA (or PA) and various groups. Figure 2c depicts a geometric comparison among the S_1_, T_1_, and T_2_, and the structure deviation are mostly originated from the DA1 and DA2.

### Singlet excited-state potential energy surface by twisting TPA or PA group

The results with non-restricted optimized S_1_, T_1_, and T_2_ structures are consistent with the hot-exciton RISC path proposed by Ma et al. However, upon the excited states (S_1_, T_1_, and T_2_), the stable CT state formation by twisting TPA or PA would affect the T_2_ and S_1_ gap and RISC process. On one hand, if the CT state is the most stable emission state, the decrease of the S_1_ state would affect the quantum efficiency by decreasing the fluorescence decay rate and aggravating the non-radiative relaxation. On the other hand, the enlarging S_1_ and T_2_ gap originated from the decreased S_1_ energy level would weaken the T_2_ exciton utilization. Like-TADF molecules based on donor and acceptor groups through a single bond, the molecular rotation was easily activated and would generate a large spectrum of conformations at room temperature^43^. For **1**, **2**, and **3** (Fig. 2a), the donor and acceptor groups are connected via a flexible single bond, therefore, the S_1_, T_1_, and T_2_ CT states by twisting TPA or PA were calculated.

At first, we performed the triplet excited-state (T_1_ and T_2_) potential energy surface (PES) (in Figure S2) with twisting TPA or PA. It can be found that almost all T_1_ and T_2_ energies are increased with twisting TPA or PA, meaning that molecular rotations are difficult to realize upon T_1_ and T_2_ PES. Consequently, the T_1_ and T_2_ energy levels are almost constant. Further, the singlet excited-state potential energy surface (S_1_ PES) with twisting TPA or PA with a scan step of 10 degrees has been performed.

**Figure 3:**
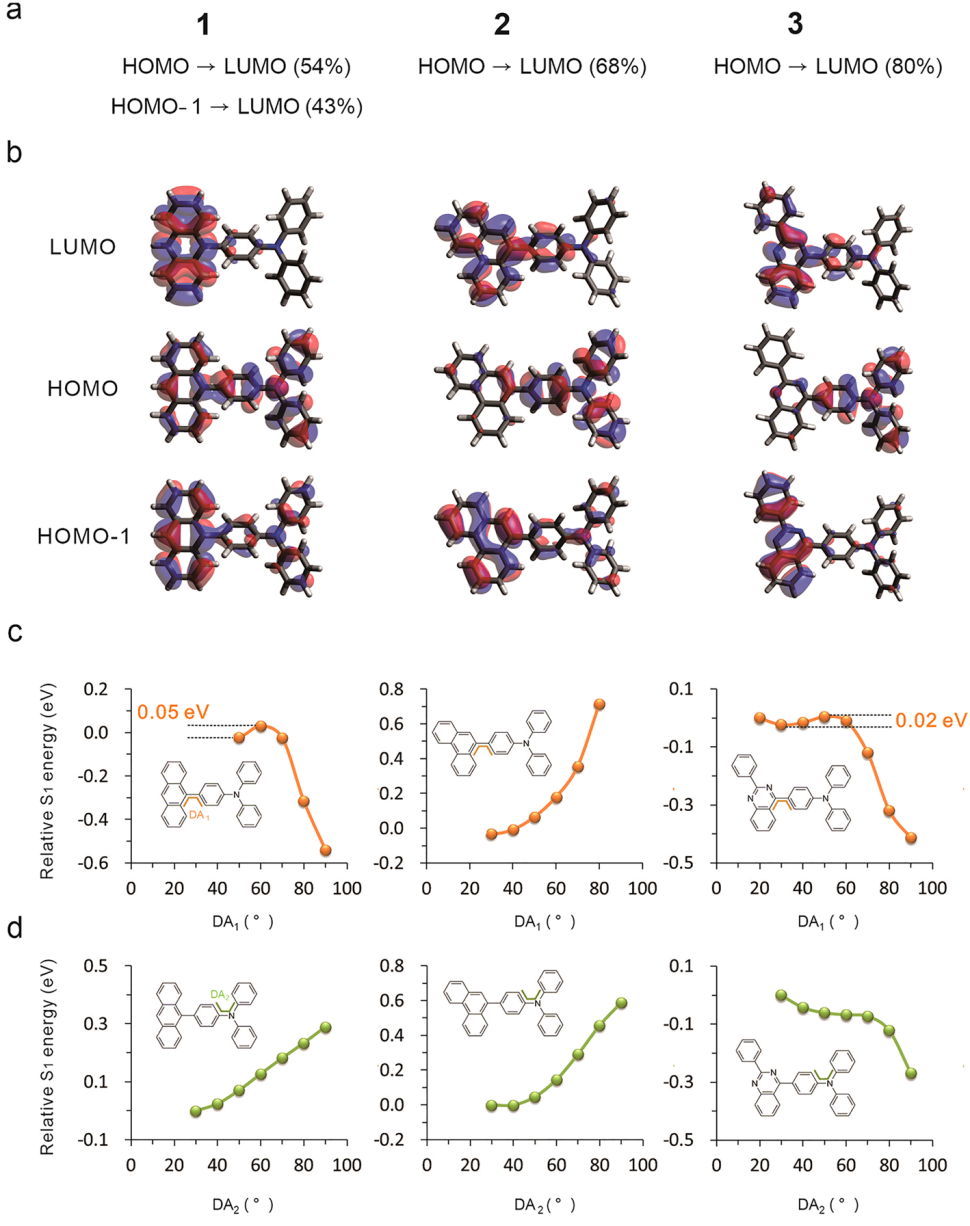
**(a)** S_1_ transition configuration of **1**, **2**, and **3**, **(b)** HOMO-1, HOMO and LUMO distribution, **(c)** S_1_ PES by twisting TPA, **(d)** S_1_ PES by twisting PA.

The vertical S_1_ states of three HLCT-state molecules based on S_0_ structures (in Table S5) display large oscillator strength in a range from 0.3560 to 0.9085, indicating main LE transition and high fluorescence emission. Figure 3b depicts the highest occupied molecular orbital (HOMO) and lowest unoccupied molecular orbital (LUMO). All S_1_ states are attributed to a mixture of more HOMO → LUMO and less HOMO-1 → LUMO contributions (Fig. 3a). For example, **3** possesses a total HOMO → LUMO (80%) transition with HOMO mainly diffused over TPA and LUMO localized on the 2-phenylquinazoline group (Fig. 3b). The HOMO-1 of **1** is distributed over the whole molecules, and the HOMO-1 → LUMO transition presents the main LE characteristic. The S_1_ states of **1**, **2**, and **3** molecules display a combination of CT and LE features.

In terms of twisting TPA (DA_1_) or PA (DA_2_) (Fig. 3c,d), the plots of S_1_ PES show three variation tendencies. It can be concluded that the CT state of **3** is very easy to realize by twisting TPA or PA. But the TPA or PA of **2** is very different to twist on the S_1_ PES. The CT state formation of **1** is in a barrierless process by twisting TPA, but not for PA. To illustrate the excited-state properties, Table S6-S13 lists the oscillator strength, transition configuration, and frontier molecular orbitals of **1**, **2**, and **3** with twisting TPA or PA.

On the S_1_ PES of **1** and **3** with twisting TPA, the CT state is more stable than the HLCT state and would become the finally stable emission state, suppressing small energy barriers (0.05 eV for **1** and 0.02 eV for **3**). At small DA_1_, the S_1_ states of **1** contain a main HOMO → LUMO transition, but a mixture of HOMO → LUMO and HOMO-1 → LUMO transitions at 70°—80° is found. With increasing DA_1_, the HOMO shifts from the whole molecules to the TPA group, and LUMO moves from the anthracene and the middle phenyl ring to the whole anthracene (Table S8). Finally, the S_1_ state (DA_1_ = 90 degrees) is a total CT character with a low f (nearly zero). The S_1_ states of **3** are assigned to HOMO → LUMO in a percentage range of 20% → 90% with increased DA_1_. Considering orbital distribution (Table S10), the initial HOMO shows a high degree of localization of electron density on the whole molecule, and transfers to TPA with increasing DA_1_. Upon the TPA rotation process, LUMO is always distributed on the 2-phenylquinazoline and the middle phenyl ring. When DA_1_ is 90 degrees, a noticeably separated HOMO and LUMO for CT state is formed, and f is nearly approaching to zero. However, the S_1_ energies of **2** are increasing with twisting TPA (Fig. 3c). The energy is the highest point on the S_1_ PES (DA_1_ = 90 degrees), indicating that TPA rotation of **2** is restrained. Therefore, the relative S_1_ and T_1_ energy levels are almost unchanged and have no influence on the T_2_ → S_1_ RISC process. Different from other HLCT molecules, the S_1_ states have large f values in a range from 0.991 to 1.433 (Table S6). With twisting TPA group, the transition configurations (Table S9) change from HLCT to total LE, accompanied by high f.

As with twisting PA (Fig. 3d), the relative energies of **1** and **2** increase linearly with increasing DA_2_, indicating that the PA rotation of **1** and **2** on S_1_ PES need to overcome the high energy barrier to realize. The S_1_ states of **1** contain a major HOMO → LUMO (Table S7). As with twisting PA, the HOMO is initially delocalized over the whole molecules and shifts to the anthracene and middle phenyl ring, and the LUMO show almost complete localization on the anthracene and middle phenyl ring. Finally, the S_1_ state (DA_2_ = 90 degrees) of **1** contains the main LE state and has non-zero f (Table S6). The S_1_ states of **2** contain a major HOMO → LUMO and minor HOMO-1 → LUMO. Along twisting PA, the HOMO and HOMO-1 are always localized over the whole molecules, and LUMO is distributed on the phenanthrene and middle phenyl ring (Table S12). When the DA_2_ is 90 degrees, the S_1_ state displays the main LE characteristic and large f (Table S6). With increasing DA_2_, the S_1_ energy of **3** is decreasing, and a stable CT state would form in a barrierless process by twisting PA. The HOMO → LUMO (84–90%) transition gives dominant contributions to S_1_ states along twisting PA. At small DA_2_, the HOMO is mostly distributed on the TPA and a small part on the 2-phenylquinazoline but shift to the TPA group with increasing DA_2_ (Table S13). When the DA_2_ is 90 degrees, the HOMO is centered on the PA group, while LUMO are distributed on 2-phenylquinazoline and middle phenyl ring with f nearly to zero for S_1_ state.

In the excited state, the CT states (DA_1_ = 90 degrees) of **1** and **3** (DA_1_/DA_2_ = 90 degrees) are easy to populate via twisting TPA or PA, accomplished by decreased f and S_1_ emission efficiency. Moreover, the formation of stable CT states would enlarge the S_1_ and T_2_ gap, weaken the T_2_ to S_1_ RISC and decrease the triplet exciton utilization.

## Conclusion

In conclusion, taking molecules **1**, **2**, and **3** as examples, we have investigated the excited-state potential energy surface (PES) by twisting triphenylamine (TPA) or diphenylamine (PA) by density functional theory and time-dependent density functional theory calculations. The calculated relative total energy indicated that the T_2_ and T_1_ energy gap is large, and T_2_ is closed to S_1_ in energy levels under the case of non-restricted excited-state optimizations. Meanwhile, the S_1_ states of **1**, **2**, and **3** have a mixture of locally excited (LE) and charge-transfer (CT) characteristics, high oscillator strength, and high fluorescence emission efficiency. Considering the excited state PES, the T_1_ and T_2_ energy levels are almost constant. However, for the S_1_ PES, it can be found that twisting TPA or PA would stabilize the CT state, which would seriously damage the quantum efficiency. For example, the CT states of **3** are very easy to realize no matter by twisting TPA or PA. The stable CT state formation of **3** would enlarge the energy gap between S_1_ and T_2_ and decrease the T_2_ exciton utilization. However, the TPA or PA group of **2** is very different to twist in the S_1_ PES, and S_1_ and T_2_ energy gap is almost unchanged. For **1**, the CT states are formed in a barrierless process by twisting TPA, but not for PA. Therefore, understanding the relationship between the S_1_ PES and molecular structures is important for designing high-performance luminescent materials utilizing HLCT state.

## Supplementary Information

Supplementary Information.

## Data Availability

The authors declare that the data and manuscript are availability.

## References

[1] Tang CW, VanSlyke SA (1987). Organic electroluminescent diodes. Appl. Phys. Lett..

[2] Baldo MA, O'Brien DF, You Y, Shoustikov A, Sibley S, Thompson ME, Forrest SR (1998). Highly efficient phosphorescent emission from organic electroluminescent devices. Nature.

[3] Baldo MA, Lamansky S, Burrows PE, Thompson ME, Forrest SR (1999). Very high-efficiency green organic light-emitting devices based on electrophosphorescence. Appl. Phys. Lett..

[4] Chen XK, Kim D, Brédas JL (2018). Thermally activated delayed fluorescence (TADF) path toward efficient electroluminescence in purely organic materials: Molecular level insight. Acc. Chem. Res..

[5] Uoyama H, Goushi K, Shizu K, Nomura H, Adachi C (2012). Highly efficient organic light-emitting diodes from delayed fluorescence. Nature.

[6] Yang ZY, Mao Z, Xie ZL, Zhang Y, Liu SW, Zhao J, Xu JR, Chi ZG, Aldred MP (2017). Recent advances in organic thermally activated delayed fluorescence materials. Chem. Soc. Rev..

[7] Li WJ, Liu DD, Shen FZ, Ma DG, Wang ZM, Feng T, Xu YX, Yang B, Ma YG (2012). A twisting donor-acceptor molecule with an intercrossed excited state for highly efficient, deep-blue electroluminescence. Adv. Funct. Mater..

[8] Li WJ, Pan YY, Xiao R, Peng QM, Zhang ST, Ma DG, Li F, Shen FZ, Wang YH, Yang B, Ma YG (2014). Employing ∼100% excitons in OLEDs by utilizing a fluorescent molecule with hybridized local and charge-transfer excited state. Adv. Funct. Mater..

[9] Han X, Bai Q, Yao L, Liu HC, Gao Y, Li JY, Liu LQ, Liu YL, Li XX, Lu P, Yang B (2015). Highly efficient solid-state near-infrared emitting material based on triphenylamine and diphenylfumaronitrile with an EQE of 2.58% in nondoped organic light-emitting diode. Adv. Funct. Mater..

[10] Li BJ, Zhou LS, Cheng H, Huang Q, Lan JB, Zhou L, You JS (2018). Dual-emissive 2-(2′-hydroxyphenyl)oxazoles for high performance organic electroluminescent devices: Discovery of a new equilibrium of excited state intramolecular proton transfer with a reverse intersystem crossing process. Chem. Sci..

[11] Zhou CJ, Gong DL, Gao Y, Liu HC, Li JY, Zhang ST, Su Q, Wu QL, Yang B (2018). Enhancing the electroluminescent efficiency of acridine-based donor-acceptor materials: Quasi-equivalent hybridized local and charge-transfer state. J. Phys. Chem. C.

[12] Liu B, Yu ZW, He D, Zhu ZL, Zheng J, Yu YD, Xie WF, Tong QX, Lee CS (2017). Ambipolar D-A type bifunctional materials with hybridized local and charge-transfer excited state for high performance electroluminescence with EQE of 7.20% and CIEy ∼ 0.06. J. Mater. Chem. C.

[13] Li C, Hanif M, Li XL, Zhang ST, Xie ZQ, Liu LL, Yang B, Su SJ, Ma YG (2016). Effect of cyano-substitution in distyrylbenzene derivatives on their fluorescence and electroluminescence properties. J. Mater. Chem. C.

[14] Konidena RK, Thomas KRJ, Dubey DK, Sahoo S, Jou JH (2017). A new molecular design based on hybridized local and charge transfer fluorescence for highly efficient (>6%) deep-blue organic light emitting diodes. Chem. Commun..

[15] Yang B, Kim SK, Xu H, Park YI, Zhang HY, Gu C, Shen FZ, Wang CL, Liu DD, Liu XD, Hanif M, Tang S, Li WJ, Li F, Shen JC, Park JW, Ma YG (2008). The origin of the improved efficiency and stability of triphenylamine-substituted anthracene derivatives for OLEDs: A theoretical investigation. ChemPhysChem.

[16] Li WJ, Pan YY, Yao L, Liu HC, Zhang ST, Wang C, Shen FZ, Lu P, Yang B, Ma YG (2014). A hybridized local and charge-transfer excited state for highly efficient fluorescent OLEDs: Molecular design, spectral character, and full exciton utilization. Adv. Opt. Mater..

[17] Zhang ST, Li W, Yao L, Pan YY, Shen FZ, Xiao R, Yang B, Ma YG (2013). Enhanced proportion of radiative excitons in non-doped electro-fluorescence generated from an imidazole derivative with an orthogonal donor–acceptor structure. Chem. Commun..

[18] Yao L, Zhang ST, Wang R, Li WJ, Shen FZ, Yang B, Ma YG (2014). Highly efficient near-infrared organic light-emitting diode based on a butterfly-shaped donor-acceptor chromophore with strong solid-state fluorescence and a large proportion of radiative excitons. Angew. Chem. Int. Ed..

[19] Zhang ST, Yao L, Peng QM, Li WJ, Pan YY, Xiao R, Gao Y, Gu C, Wang ZM, Lu P, Li F, Su SJ, Yang B, Ma YG (2015). Achieving a significantly increased efficiency in nondoped pure blue fluorescent OLED: A quasi-equivalent hybridized excited state. Adv. Funct. Mater..

[20] Liu HC, Bai Q, Yao L, Zhang HY, Xu H, Zhang ST, Li WJ, Gao Y, Li JY, Lu P, Wang HY, Yang B, Ma YG (2015). Highly efficient near ultraviolet organic light-emitting diode based on a meta-linked donor–acceptor molecule. Chem. Sci..

[21] Xu YW, Liang XM, Zhou XH, Yuan PS, Zhou JD, Wang C, Li BB, Hu DH, Qiao XF, Jiang XF, Liu LL, Su SJ, Ma DG, Ma YG (2019). Highly efficient blue fluorescent OLEDs based on upper level triplet-singlet intersystem crossing. Adv. Mater..

[22] Chen LF, Zhang ST, Li H, Chen RF, Jin L, Yuan K, Li HH, Lu P, Yang B, Huang W (2018). Breaking the efficiency limit of fluorescent OLEDs by hybridized local and charge-transfer host materials. J. Phys. Chem. Lett..

[23] Li XB, Lv YN, Chang SY, Liu HQ, Mo WQ, Ma HW, Zhou CJ, Zhang ST, Yang B (2019). Visualization of ultrasensitive and recyclable dual-channel fluorescence sensors for chemical warfare agents based on the state dehybridization of hybrid locally excited and charge transfer materials. Anal. Chem..

[24] Pan YY, Li WJ, Zhang ST, Yao L, Gu C, Xu H, Yang B, Ma YG (2014). High yields of singlet excitons in organic electroluminescence through two paths of cold and hot excitons. Adv. Opt. Mater..

[25] Pan YY, Huang J, Wang ZM, Zhang ST, Yu DW, Yang B, Ma YG (2016). Accurate description of hybridized local and charge-transfer excited-state in donor–acceptor molecules using density functional theory. RSC Adv..

[26] Jiang YR, Hu ZB, Zhou B, Zhong C, Sun ZR, Sun HT (2019). Accurate prediction for dynamic hybrid local and charge transfer excited states from optimally tuned range-separated density functionals. J. Phys. Chem. C.

[27] Kautny P, Glöcklhofer F, Kader T, Mewes JM, Stöger B, Fröhlich J, Lumpi D, Plasser F (2017). Charge-transfer states in triazole linked donor–acceptor materials: Strong effects of chemical modification and solvation. Phys. Chem. Chem. Phys..

[28] Zhou CJ, Zhang XY, Pan GC, Tian XZ, Xiao SB, Liu HC, Zhang ST, Yang B (2019). Investigation on excited-state properties and electroluminescence performance of donor−acceptor materials based on quinoxaline derivatives. Org. Electron..

[29] Li YX, Tan TF, Wang ST, Xiao Y, Li XG (2017). Highly solvatochromic fluorescence of anthraquinone dyes based on triphenylamines. Dyes Pigments.

[30] Shen Y, Tang XH, Xu YW, Liu HC, Zhang ST, Yang B, Ma YG (2019). Enhanced deep-red emission in donor-acceptor molecular architecture: The role of ancillary acceptor of cyanophenyl. Chin. Chem. Lett..

[31] Song HW, Wang K, Kuang ZR, Zhao YS, Guo QJ, Xia AD (2019). Solvent modulated excited state processes of push–pull molecule with hybridized local excitation and intramolecular charge transfer character. Phys. Chem. Chem. Phys..

[32] Fan D, Yi YP, Li ZD, Liu WJ, Peng Q, Shuai ZG (2015). Solvent effects on the optical spectra and excited-state decay of triphenylamine-thiadiazole with hybridized local excitation and intramolecular charge transfer. J. Phys. Chem. A.

[33] Li JW, Qian Y, Xie LH, Yi YP, Li WW, Huang W (2015). From dark TICT state to emissive quasi-TICT state: The AIE mechanism of N-(3-(benzo[d]oxazol-2-yl)phenyl)-4-tert-butylbenzamide. J. Phys. Chem. C.

[34] Hu TP, Han GC, Tu ZY, Duan RH, Yi YP (2018). Origin of high efficiencies for thermally activated delayed fluorescence organic light-emitting diodes: Atomistic insight into molecular orientation and torsional disorder. J. Phys. Chem. C.

[35] Moral M, Son WJ, Sancho-García JC, Oivier Y, Muccioli L (2015). Cost-effective force field tailored for solid-phase simulations of OLED materials. J. Chem. Theory Comput..

[36] Olivier Y, Yurash B, Muccioli L, D’Avino G, Mikhnenko O, Sancho-García JC, Adachi C, Nguyen TQ, Beljonne D (2017). Nature of the singlet and triplet excitations mediating thermally activated delayed fluorescence. Phys. Rev. Mater..

[37] Peach MJG, Williamson MJ, Tozer DJ (2011). Influence of triplet instabilities in TDDFT. J. Chem. Theory Comput..

[38] Hirata S, Head-Gordon M (1999). Time-dependent density functional theory within the Tamm-Dancoff approximation. Chem. Phys. Lett..

[39] Caricato M, Mennucci B, Tomasi J, Ingrosso F, Cammi R, Corni S, Scalmani G (2006). Formation and relaxation of excited states in solution: A new time dependent polarizable continuum model based on time dependent density functional theory. J. Chem. Phys..

[40] Marenich AV, Cramer CJ, Truhlar DG (2009). Universal solvation model based on solute electron density and on a continuum model of the solvent defined by the bulk dielectric constant and atomic surface tensions. J. Phys. Chem. B.

[41] Frisch MJ, Trucks GW, Schlegel HB, Scuseria GE, Robb MA, Cheeseman JR, Scalmani G, Barone V, Petersson GA, Nakatsuji H, Li X, Caricato M, Marenich AV, Bloino J, Janesko BG, Gomperts R, Mennucci B, Hratchian HP, Ortiz JV, Izmaylov AF, Sonnenberg JL, Williams-Young D, Ding F, Lipparini F, Egidi F, Goings J, Peng B, Petrone A, Henderson T, Ranasinghe D, Zakrzewski VG, Gao J, Rega N, Zheng G, Liang W, Hada M, Ehara M, Toyota K, Fukuda R, Hasegawa J, Ishida M, Nakajima T, Honda Y, Kitao O, Nakai H, Vreven T, Throssell K, Montgomery JA, Peralta JE, Ogliaro F, Bearpark MJ, Heyd JJ, Brothers EN, Kudin KN, Staroverov VN, Keith TA, Kobayashi R, Normand J, Raghavachari K, Rendell AP, Burant JC, Iyengar SS, Tomasi J, Cossi M, Millam JM, Klene M, Adamo C, Cammi R, Ochterski JW, Martin RL, Morokuma K, Farkas O, Foresman JB, Fox DJ (2016). Gaussian 16, Revision A.03.

[42] Lu T, Chen FW (2012). Multiwfn: A multifunctional wavefunction analyzer. J. Comput. Chem..

[43] Olivier Y, Sancho-Garcia JC, Muccioli L, D’Avino G, Beljonne D (2018). Computational design of thermally activated delayed fluorescence materials: The challenges ahead. J. Phys. Chem. Lett..

