# Supplementary material for: Effect of hybridized local and charge transfer molecules rotation in excited state on exciton utilization
Source: Sci Rep. 2021 Sep 3;11:17686. doi: 10.1038/s41598-021-97229-z (PMC8417272; doi:10.1038/s41598-021-97229-z)
Supplement: Supplementary file 1 — Supplementary Information. [file 41598_2021_97229_MOESM1_ESM.doc]

Supporting Information

**Effect of Hybridized Local and Charge transfer Molecules Rotation in Excited State on Exciton Utilization**

Gang Sun^a^, Xin-Hui Wang^a^, Jing Li^a^, Bo-Ting Yang^a^,* Ying Gao^b^ **, Yun Geng^c^

*^a^ College of Science, Beihua University, Jilin 132013, China*

*^b^ Jilin Provincial Key Laboratory of Straw-Based Functional Materials, Institute for Interdisciplinary Biomass Functional Materials Studies, Jilin Engineering Normal University, Changchun 130052, P. R. China*

*^c^ Faculty of Chemistry, Northeast Normal University, Changchun 130024, China*

*Corresponding author. E-mail: [ybt199@126.com](mailto:ybt199@126.com);

** Corresponding author. E-mail: gaoy029@163.com;

Table S1 Calculated S_1_ excited-state parameters using three methods based S_0_ structures.

| M062X | S_1_ | S_r_ (au) | D (Å) | H (Å) | t (Å) |
| --- | --- | --- | --- | --- | --- |
| **1** | 3.452 | 0.864 | 0.671 | 3.343 | -1.557 |
| **2** | 3.990 | 0.750 | 2.161 | 3.948 | -1.317 |
| **3** | 3.561 | 0.643 | 3.392 | 3.576 | 0.457 |
| WB97XD | S_1_ | S_r_ (au) | D (Å) | H (Å) | t (Å) |
| **1** | 3.472 | 0.889 | 0.225 | 3.116 | -1.668 |
| **2** | 4.111 | 0.788 | 1.317 | 4.043 | -2.281 |
| **3** | 3.737 | 0.709 | 2.342 | 3.659 | -0.715 |
| CAM-B3LYP | S_1_ | S_r_ (au) | D (Å) | H (Å) | t (Å) |
| **1** | 3.443 | 0.882 | 0.385 | 3.218 | -1.649 |
| **2** | 4.041 | 0.776 | 1.728 | 4.045 | -1.864 |
| **3** | 3.630 | 0.672 | 3.014 | 3.596 | 0.044 |

Table S2 Calculated T_1_ excited-state parameters using three methods based on S_0_ structures.

| M062X | T_1_ | S_r_ (au) | D (Å) | H (Å) | t (Å) |
| --- | --- | --- | --- | --- | --- |
| **1** | 2.189 | 0.898 | 0.051 | 2.913 | -1.506 |
| **2** | 3.098 | 0.907 | 0.354 | 3.520 | -2.559 |
| **3** | 2.913 | 0.825 | 1.117 | 3.813 | -2.039 |
| WB97XD | T_1_ | S_r_ (au) | D (Å) | H (Å) | t (Å) |
| **1** | 1.733 | 0.915 | 0.026 | 2.859 | -1.471 |
| **2** | 2.636 | 0.944 | 0.080 | 3.118 | -2.221 |
| **3** | 2.660 | 0.897 | 0.164 | 3.557 | -2.291 |
| CAM-B3LYP | T_1_ | S_r_ (au) | D (Å) | H (Å) | t (Å) |
| **1** | 1.595 | 0.919 | 0.019 | 2.882 | -1.490 |
| **2** | 2.486 | 0.949 | 0.066 | 3.107 | -2.194 |
| **3** | 2.533 | 0.902 | 0.165 | 3.593 | -2.412 |

Table S3 Calculated T_2_ excited-state parameters using three methods based on S_0_ structures.

| M062X | T_2_ | S_r_ (au) | D (Å) | H (Å) | t (Å) |
| --- | --- | --- | --- | --- | --- |
| **1** | 3.451 | 0.803 | 1.040 | 3.195 | -1.559 |
| **2** | 3.467 | 0.845 | 0.665 | 3.998 | -2.821 |
| **3** | 3.284 | 0.849 | 0.673 | 4.270 | -2.838 |
| WB97XD | T_2_ | S_r_ (au) | D (Å) | H (Å) | t (Å) |
| **1** | 3.188 | 0.884 | 0.411 | 3.039 | -2.009 |
| **2** | 3.171 | 0.890 | 0.404 | 3.576 | -2.623 |
| **3** | 3.052 | 0.873 | 0.650 | 4.052 | -2.741 |
| CAM-B3LYP | T_2_ | S_r_ (au) | D (Å) | H (Å) | t (Å) |
| **1** | 3.045 | 0.896 | 0.374 | 3.063 | -2.058 |
| **2** | 3.031 | 0.901 | 0.354 | 3.569 | -2.656 |
| **3** | 2.921 | 0.881 | 0.684 | 4.097 | -2.749 |

Table S4 BL1, BL2, DA1 and DA2 in the S_0_, S_1_, T_1_ and T_2_ structures.

|  | States | BL_1_ (Å) | BL12(Å) | DA_1_ (°) | DA2 (°) |
| --- | --- | --- | --- | --- | --- |
| **1** | S_0_ | 1.491 | 1.412 | -69.0 | 38.9 |
|  | S_1_ | 1.450 | 1.390 | -45.9 | 28.8 |
|  | T_1_ | 1.475 | 1.407 | -57.1 | 34.6 |
|  | T_2_ | 1.468 | 1.378 | -53.9 | 34.8 |
| **2** | S_0_ | 1.488 | 1.410 | 52.3 | -36.5 |
|  | S_1_ | 1.423 | 1.381 | 23.0 | -30.8 |
|  | T_1_ | 1.418 | 1.389 | 21.2 | -29.6 |
|  | T_2_ | 1.484 | 1.387 | 54.6 | -35.4 |
| **3** | S_0_ | 1.483 | 1.403 | -43.7 | 31.5 |
|  | S_1_ | 1.433 | 1.379 | -21.1 | 33.0 |
|  | T_1_ | 1.421 | 1.375 | -17.6 | 28.3 |
|  | T_2_ | 1.470 | 1.394 | -37.6 | 36.2 |

Table S5 Calculated S_1_ excited-state properties with M062X functional.

|  | Excited energy (eV)/  Wavelength (nm) | Oscillator  Strength | Configuration |
| --- | --- | --- | --- |
| **1** | 3.45/359 | 0.3560 | HOMO→LUMO (54%)  HOMO-1→LUMO (43%) |
| **2** | 3.99/311 | 0.9085 | HOMO→LUMO (68%)  HOMO→LUMO+3 (19%) |
| **3** | 3.56/348 | 0.7446 | HOMO→LUMO (80%) |

Table S6 Calculated oscillator strength by rotating **TPA** or **PA** group.

| **1**  DA1 |  | **2**  DA1 |  | **3**  DA1 |  |
| --- | --- | --- | --- | --- | --- |
|  |  | 20 | 1.433 | 20 | 1.217 |
|  |  | 30 | 1.425 | 30 | 1.151 |
| 40 | 0.716 | 40 | 1.397 | 40 | 1.055 |
| 50 | 0.603 | 50 | 1.329 | 50 | 0.910 |
| 60 | 0.486 | 60 | 1.217 | 60 | 0.710 |
| 70 | 0.033 | 70 | 0.940 | 70 | 0.430 |
| 80 | 0.002 | 80 | 0.9903 | 80 | 0.128 |
| 90 | 0.000 | 90 | 0.9914 | 90 | 0.000 |
| **1**  DA2 |  | **2**  DA2 |  | **3**  DA2 |  |
| 30 | 0.650 | 30 | 1.436 | 30 | 1.237 |
| 40 | 0.623 | 40 | 1.392 | 40 | 1.141 |
| 50 | 0.568 | 50 | 1.322 | 50 | 1.018 |
| 60 | 0.498 | 60 | 1.258 | 60 | 0.864 |
| 70 | 0.451 | 70 | 1.199 | 70 | 0.677 |
| 80 | 0.414 | 80 | 1.150 | 80 | 0.442 |
| 90 | 0.393 | 90 | 1.115 | 90 | 0.156 |

Table S7 S_1_ transition nature by twisting **TPA** or **PA** group.

| **1**  DA1 (°) |  |  | **2**  DA1 (°) |  |  | **3**  DA1 (°) |  |  |
| --- | --- | --- | --- | --- | --- | --- | --- | --- |
|  |  |  | 20 | H→L | 94% | 20 | H→L | 90% |
|  |  |  | 30 | H→L | 93% | 30 | H→L | 88% |
| 40 | H→L | 95% | 40 | H→L | 90% | 40 | H→L | 86% |
| 50 | H→L | 93% | 50 | H→L | 85% | 50 | H→L | 83% |
| 60 | H→L | 90% | 60 | H→L  H→L+2 | 76%  14% | 60 | H→L | 82% |
| 70 | H-1→L  H→L | 65%  26% | 70 | H→L  H→L+2 | 49%  44% | 70 | H→L | 82% |
| 80 | H-1→L  H→L | 44%  55% | 80 | H-1→L  H→L | 72%  26% | 80 | H→L | 89% |
| 90 | H→L | 93% | 90 | H-1→L | 98% | 90 | H→L | 90% |
| **1**  DA2 (°) |  |  | **2**  DA2 (°) |  |  | **3**  DA2 (°) |  |  |
|  |  |  | 30 | H→L | 94% | 30 | H→L | 90% |
| 40 | H→L | 93% | 40 | H→L | 93% | 40 | H→L | 89% |
| 50 | H→L | 91% | 50 | H→L | 92% | 50 | H→L | 88% |
| 60 | H→L | 91% | 60 | H→L | 90% | 60 | H→L | 86% |
| 70 | H→L | 93% | 70 | H-1→L  H→L | 10%  86% | 70 | H→L | 84% |
| 80 | H→L | 96% | 80 | H-1→L  H→L | 17%  80% | 80 | H→L | 83% |
| 90 | H→L | 99% | 90 | H-1→L  H→L | 22%  77% | 90 | H→L | 85% |

Table S8 HOMO-1, HOMO and LUMO distributions of **1** with **TPA** rotation.

| TPA-AN-DA1 (°) | HOMO-1 | HOMO | LUMO |
| --- | --- | --- | --- |
| 40 |  | 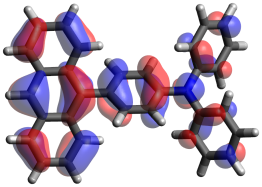 | 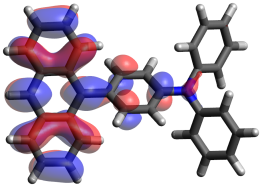 |
| 50 |  | 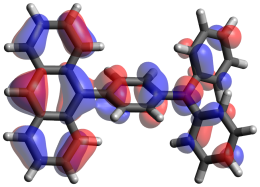 | 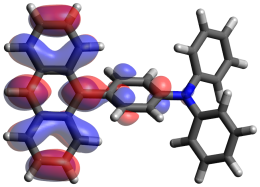 |
| 60 |  | 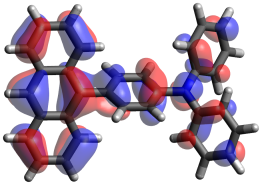 | 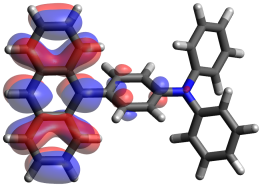 |
| 70 | 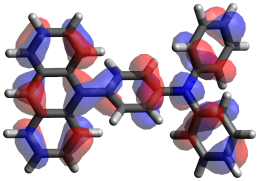 | 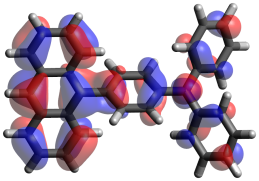 | 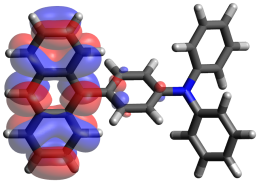 |
| 80 | 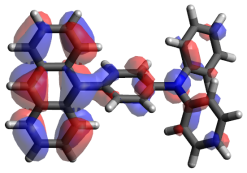 | 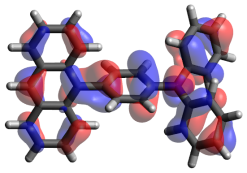 | 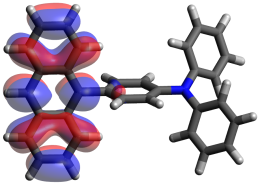 |
| 90 |  | 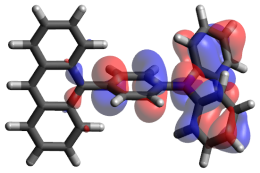 | 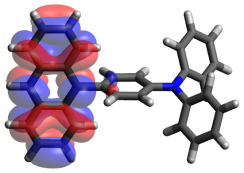 |

Table S9 HOMO-1, HOMO and LUMO distributions of **2** with **TPA** rotation

| **TPA-PA**-DA1 | LUMO+2(HOMO-1) | HOMO | LUMO |
| --- | --- | --- | --- |
| 20 |  | 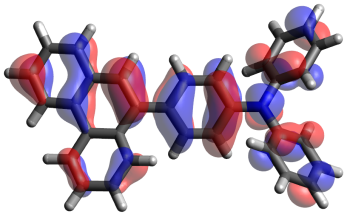 | 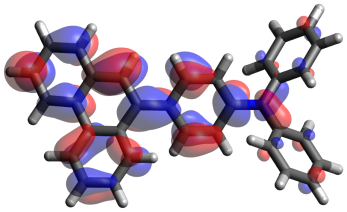 |
| 30 |  | 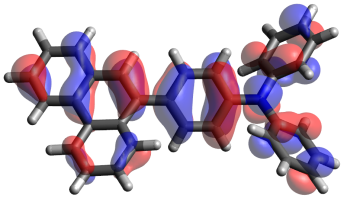 | 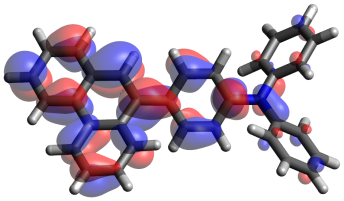 |
| 40 |  | 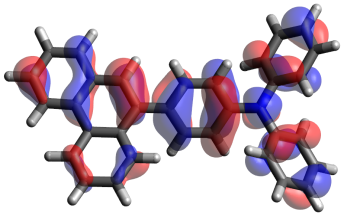 | 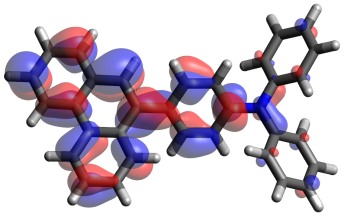 |
| 50 |  | 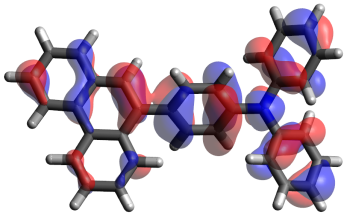 | 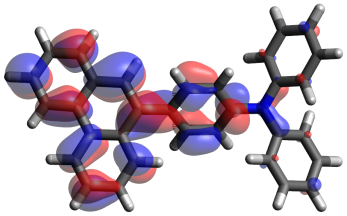 |
| 60 | 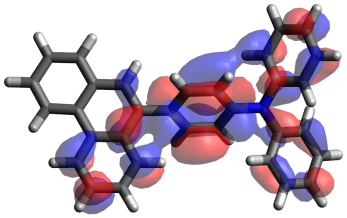 | 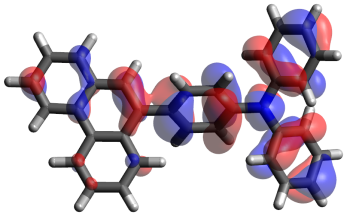 | 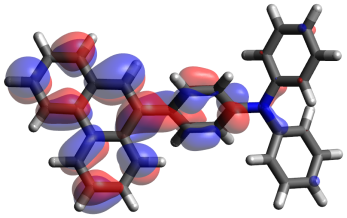 |
| 70 | 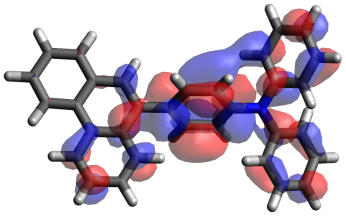 | 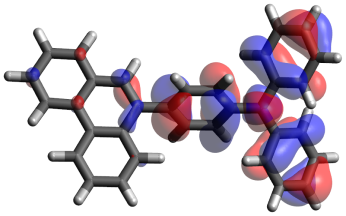 | 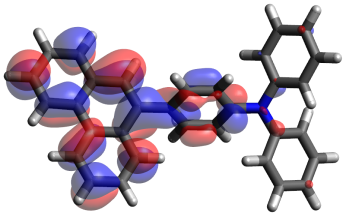 |
| 80 | 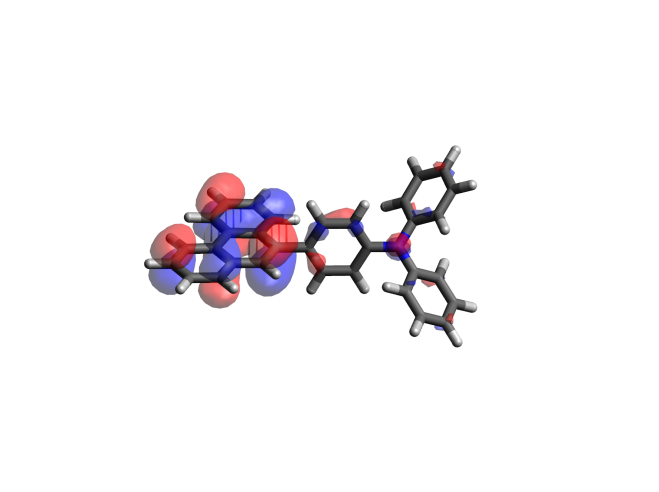 | 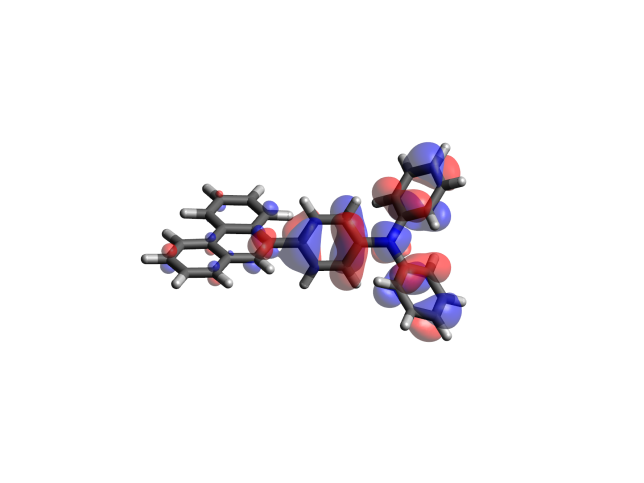 | 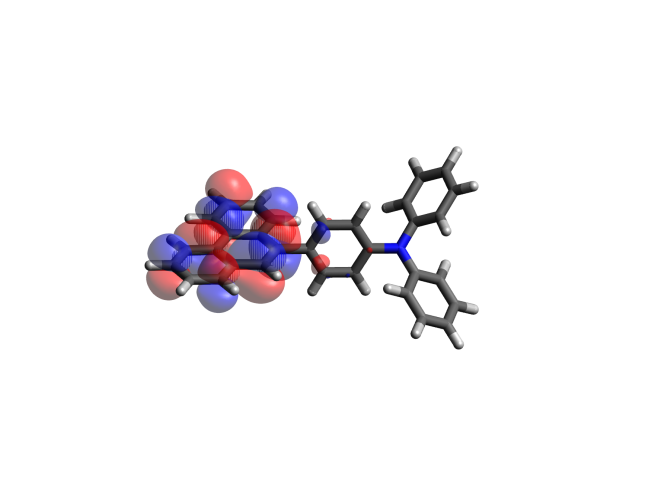 |
| 90 | 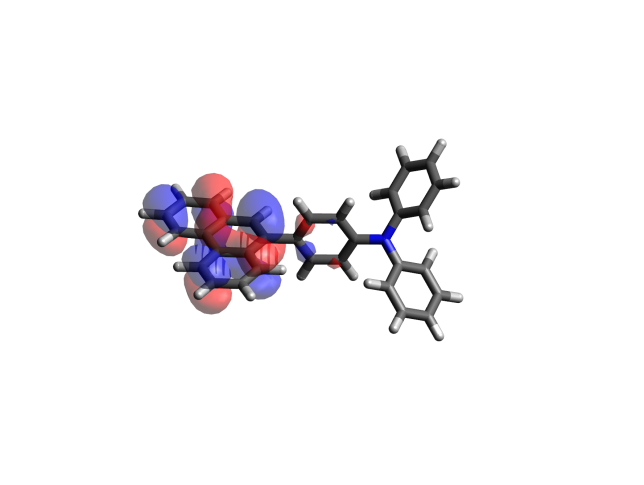 |  | 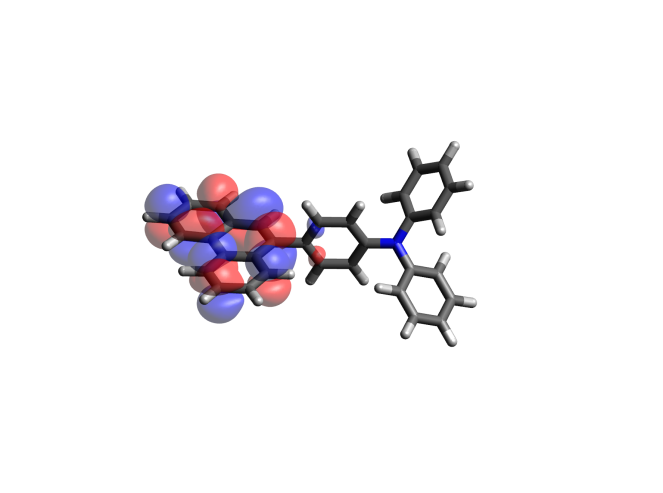 |

Table S10 HOMO and LUMO distributions of **3** with **TPA** rotation.

| **TPA-QAP**-DA1 | HOMO | LUMO |
| --- | --- | --- |
| 20 | 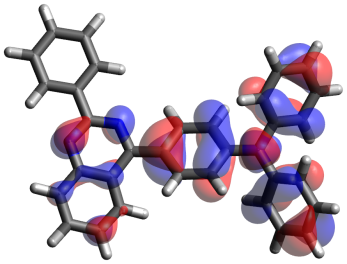 | 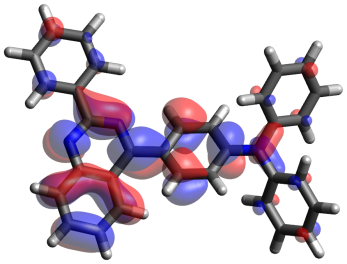 |
| 30 | 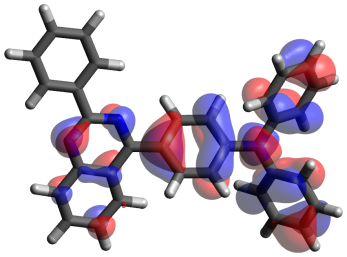 | 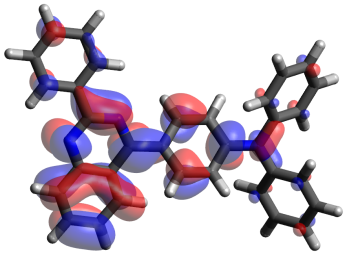 |
| 40 | 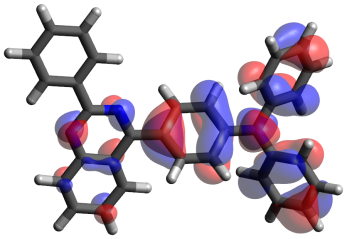 | 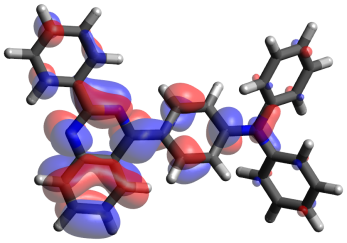 |
| 50 | 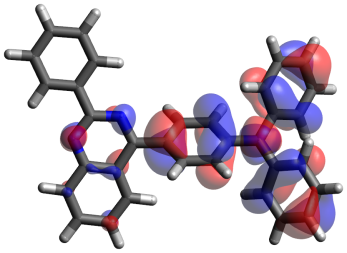 | 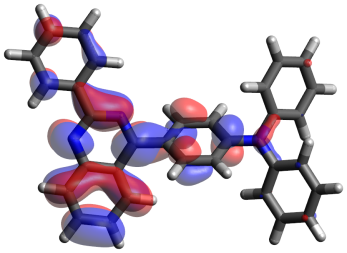 |
| 60 | 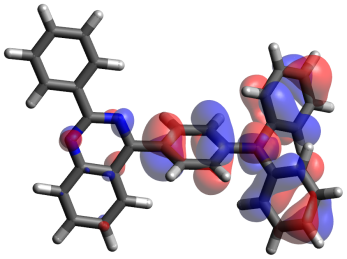 | 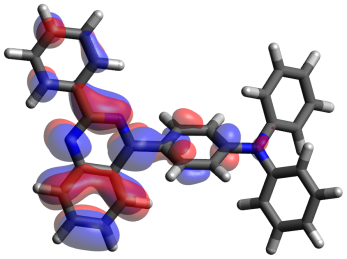 |
| 70 | 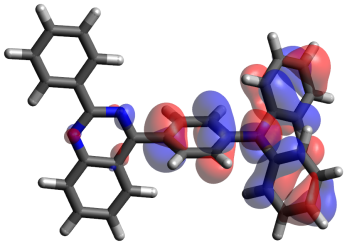 | 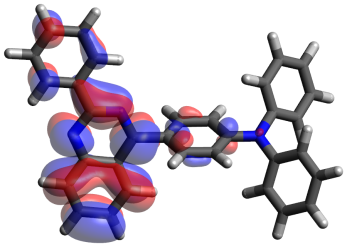 |
| 80 | 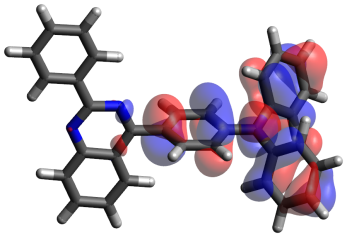 | 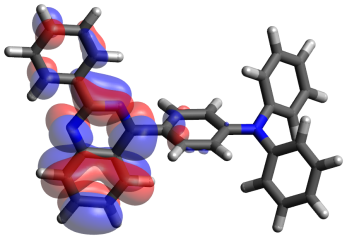 |
| 90 | 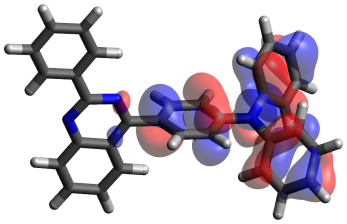 | 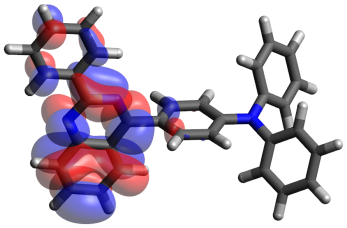 |

Table S11 HOMO and LUMO distributions of **1** with **PA** rotation.

| **TPA-AN**-DA2 (°) | HOMO | LUMO |
| --- | --- | --- |
| 40 | 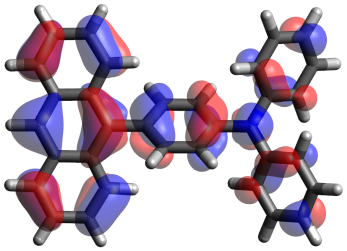 | 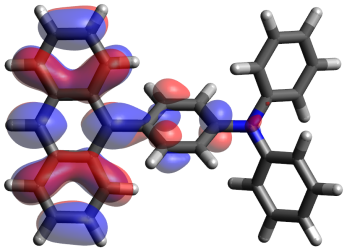 |
| 50 | 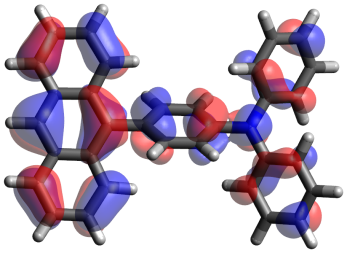 | 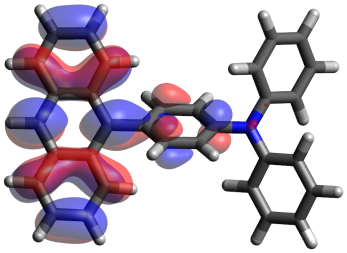 |
| 60 | 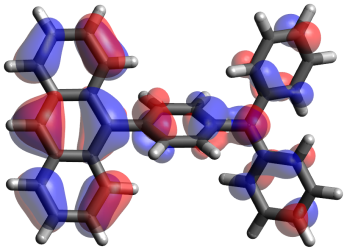 | 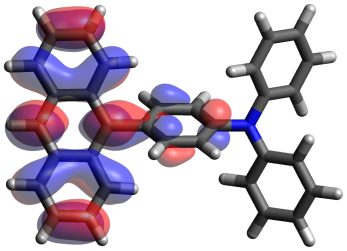 |
| 70 | 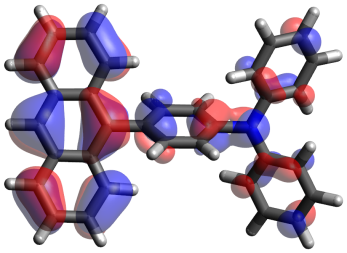 | 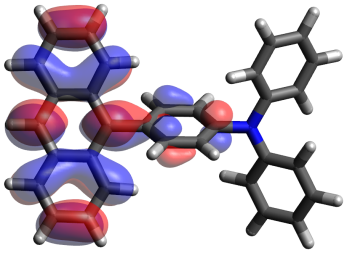 |
| 80 | 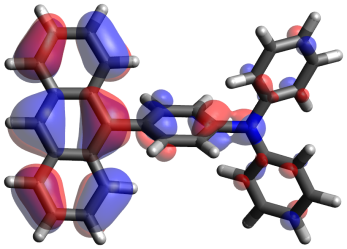 | 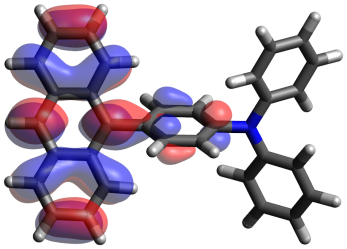 |
| 90 | 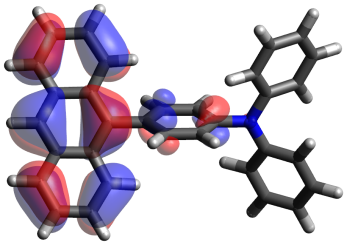 | 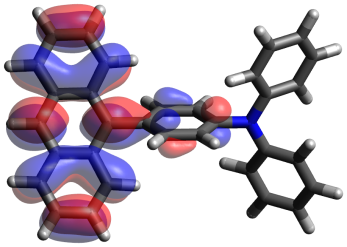 |

Table S12 HOMO-1, HOMO and LUMO distributions of **2** with **PA** rotation.

| **TPA-PA**-DA2 (°) | HOMO-1 | HOMO | LUMO |
| --- | --- | --- | --- |
| 30 |  | 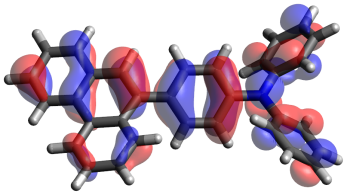 | 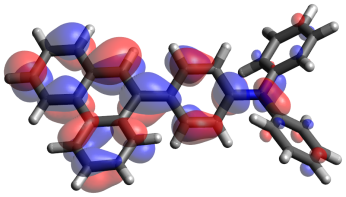 |
| 40 |  | 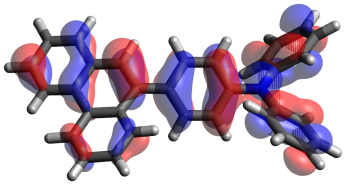 | 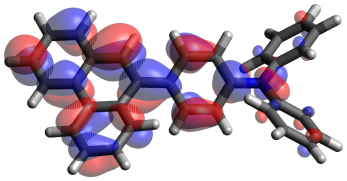 |
| 50 |  | 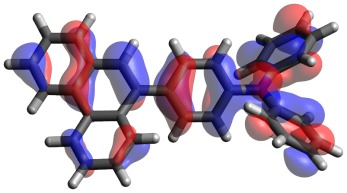 | 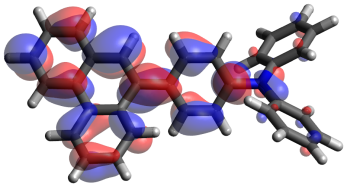 |
| 60 |  | 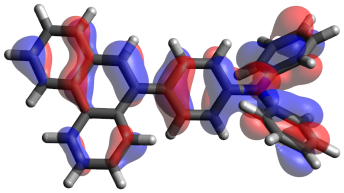 | 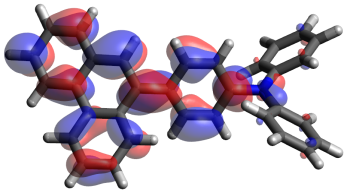 |
| 70 | 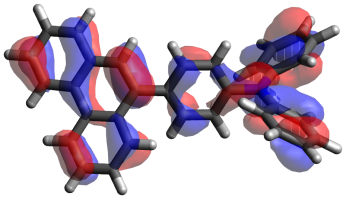 | 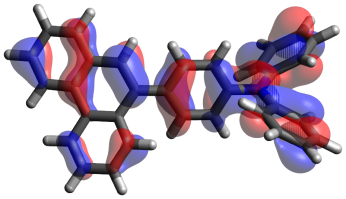 | 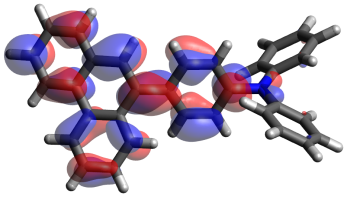 |
| 80 | 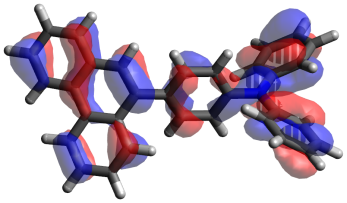 | 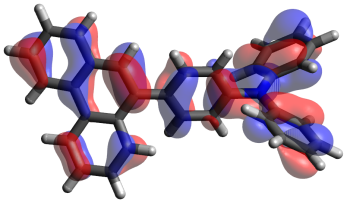 | 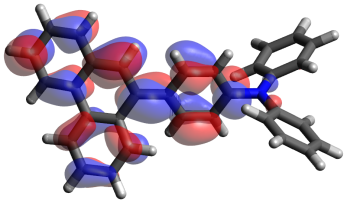 |
| 90 | 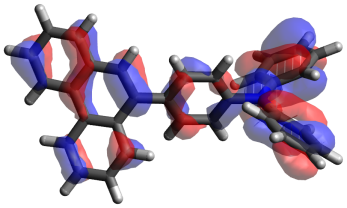 | 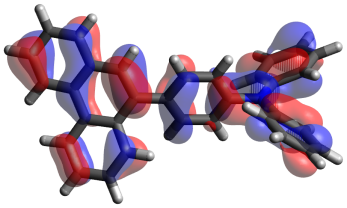 | 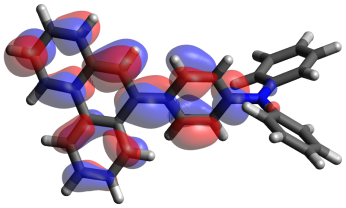 |

Table S13 HOMO and LUMO distributions of **3** with **PA** rotation.

| **TPA-QAP**-DA2 (°) | HOMO | LUMO |
| --- | --- | --- |
| 30 | 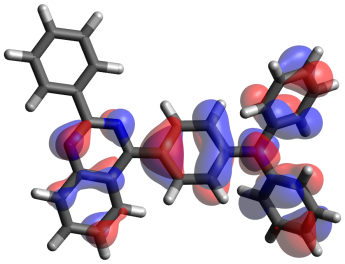 | 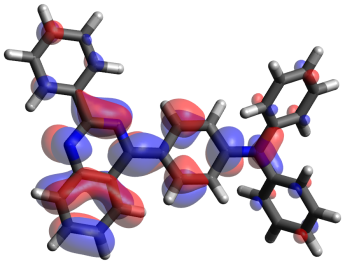 |
| 40 | 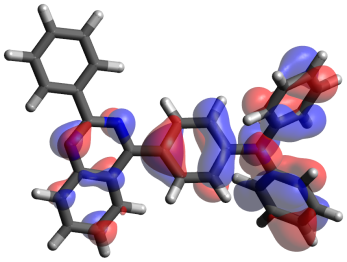 | 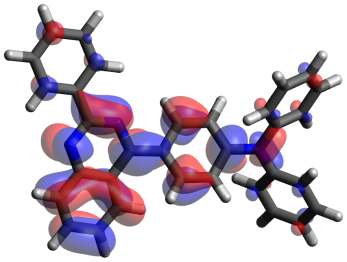 |
| 50 | 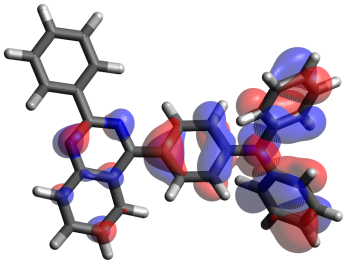 | 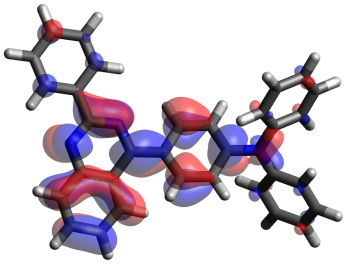 |
| 60 | 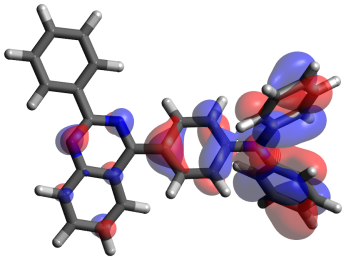 | 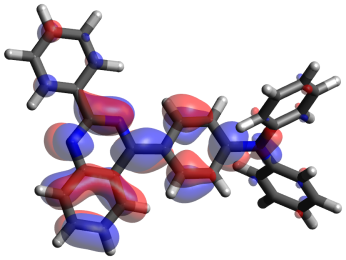 |
| 70 | 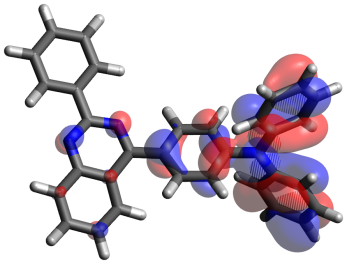 | 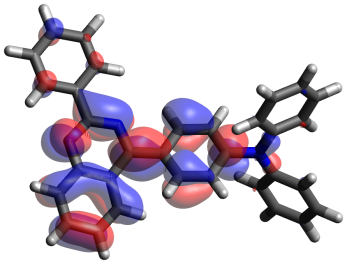 |
| 80 | 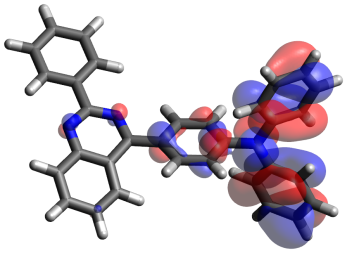 | 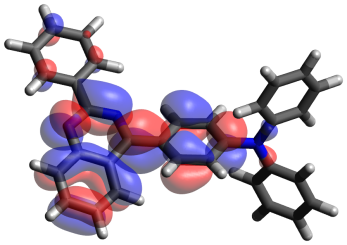 |
| 90 | 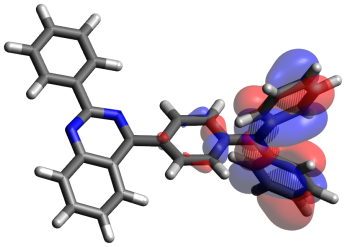 | 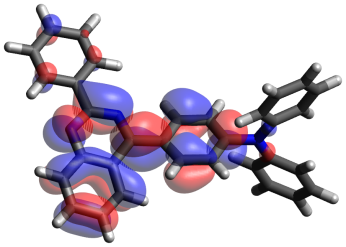 |


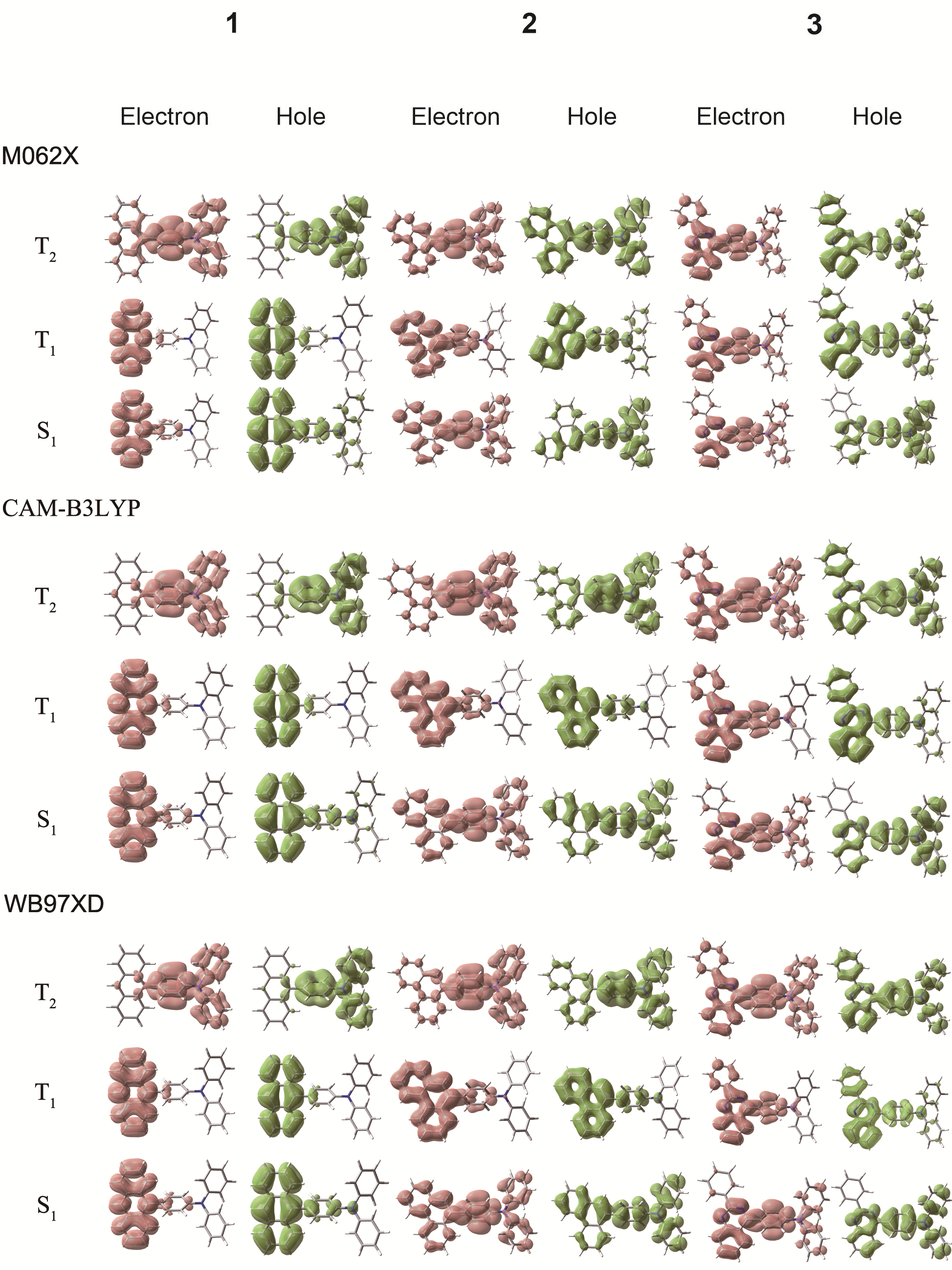


Figure S1 S1, T1 and T2 electron and hole distributions of 1, 2 and 3 with M06-2X, CAM-B3LYP and WB97XD. Figure 1S is drawn by GaussView 6.0 and Multiwfn programs;


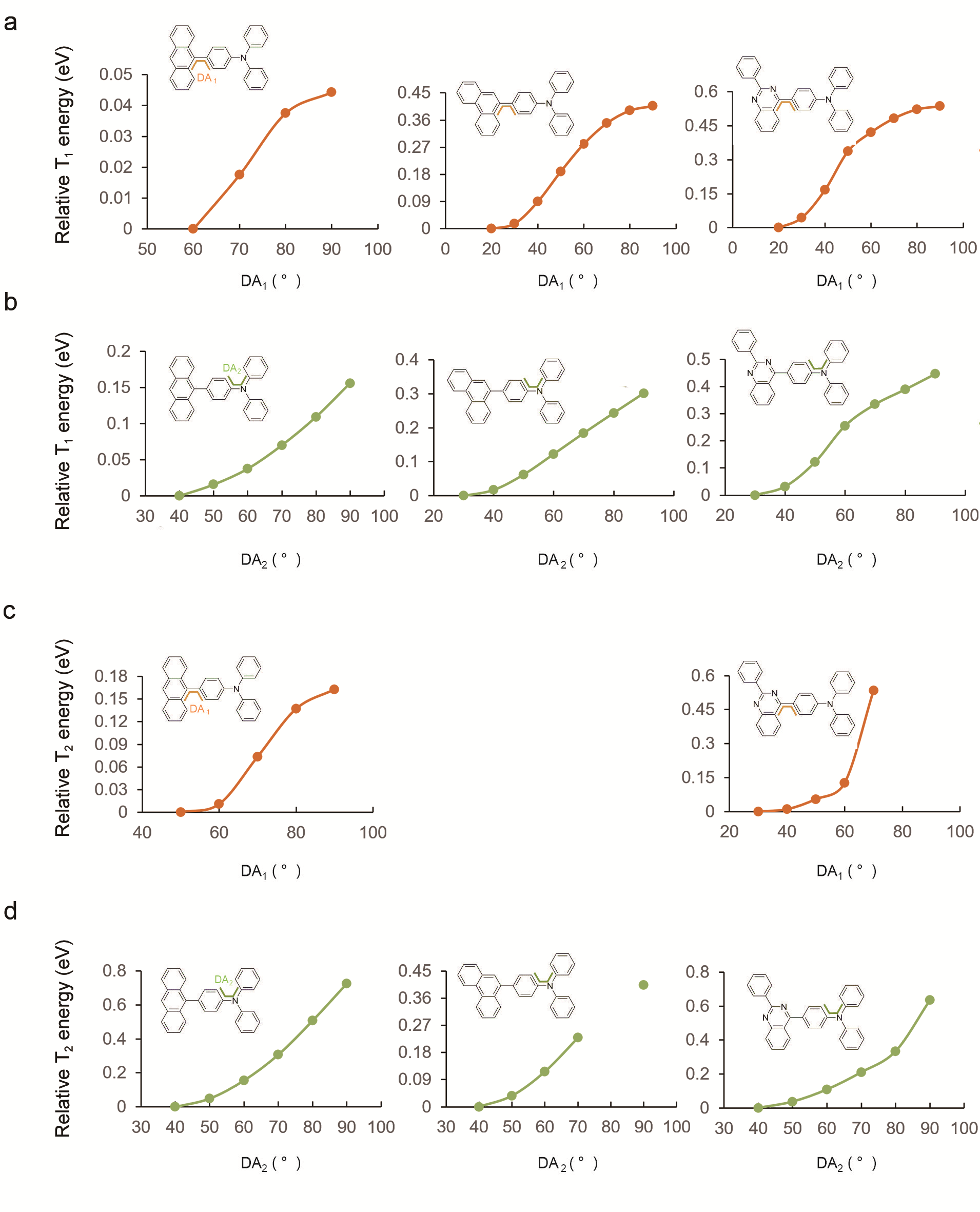


Figure S2 (a) T_1_ PES by twisting TPA, (b) T_1_ PES by twisting PA, (c) T_2_ PES by twisting TPA, (d) T_1_ PES by twisting PA. * For molecule 2, the M06-2X functional could not obtain the right T_2_ excited state when twisting TPA.
